# Supplementary material for: End-of-life care in hematological malignancies – a nationwide comparative study on the Swedish Register of Palliative Care
Source: PLoS One. 2025 Apr 29;20(4):e0312910. doi: 10.1371/journal.pone.0312910 (PMC12040083; doi:10.1371/journal.pone.0312910)
Supplement: S1 Appendix — (PDF) [file pone.0312910.s001.pdf]

English version of the questionnaire used for registering deaths in the Swedish Register of Palliative Care since January 1st 2018

1. Unit identification code ______________________

2. Personal identity number of the deceased person _______________________

3. First and last name of the deceased person _____________________________

4. Date of death (year/month/day) _________________ Time of death (hour/minute) (Optional data) ___________

5a. Date (year/month/day) when the person was admitted to the unit where the death occurred (for home care, please state the date when home care was initiated)

____

5b. Admitted from:
◻ Nursing home – permanent stay
◻ Nursing home – short-term stay
◻ Hospital ward (not hospice/palliative in-patient care)
◻ Hospice/palliative in-patient care
◻ Own home with support from specialised palliative home-care team
◻ Own home with support from general palliative home-care team
◻ Own home with daily support from home care service
◻ Own home without support
◻ Other, specify ______________________________

6. The place of death is best described as:
◻ Nursing home – permanent stay
◻ Nursing home – short-term stay
◻ Hospital ward (not hospice/palliative in-patient care)
◻ Hospice/palliative in-patient care
◻ Own home with support from specialised palliative home-care team
◻ Own home with support from general palliative home-care team
◻ Own home with daily support from home care service
◻ Own home without support
◻ Other, specify______________________________

7 a. Disease/basic state that caused the death (more than one answer is possible):
◻ Cancer
◻ Cardiovascular disease
◻ Respiratory disease
◻ Dementia
◻ Stroke
◻ Other neurological disease
◻ Diabetes

□ State after fracture
□ Multimorbidity
□ Infection
□ Other, namely: ______________________________

8. Based on the disease trajectory, was the death expected?
□ Yes □ No □ Don't know

If the answer is Yes or Don't know, answer all the following questions. If the answer is No, answer only questions 13, 15, 17, 27-29.

9. How long before death did the person lose the ability to express his/her will and take part in decisions concerning the content of medical care?
□ Retained ability until end of life
□ Hour/hours
□ Day/days
□ Week/weeks
□ Month or more
□ Has never had the ability
□ Don't know

10 a. Do the medical records include a documented decision by the physician responsible to shift treatment/care to end-of-life care?
□ Yes, in free text
□ Yes, as a classification code
□ No
□ Don't know

10 b. Did the person receive information about the transition to end-of-life care, i.e. an individually tailored and informed conversation with a physician that is documented in the medical records about being in the final stage of life and about care being focused on quality of life and symptom relief?
□ Yes
□ No
□ No, lacks the ability to participate
□ No, offered but declined
□ No, guardian opposes
□ Don't know

11. Was the person's last expressed wish about place of death known?
□ Yes □ No □ Don't know

12 a. Did the person have pressure ulcers upon arrival at your unit (specify highest category occurring)?
□ Yes, category 1
□ Yes, category 2
□ Yes, category 3
□ Yes, category 4
□ No
□ Don't know

If the answer is Yes (category 1-4), answer question 12B.
If the answer is No or Don't know, skip to question 13A.

12 b. Were the pressure ulcers documented?
□ Yes □ No □ Don't know

13 a. Did the person die with pressure ulcers (specify highest category occurring)?
□ Yes, category 1
□ Yes, category 2
□ Yes, category 3
□ Yes, category 4
□ No
□ Don't know

If the answer is Yes (category 1-4), answer question 13B.
If the answer is No or Don't know, skip to question 14A.

13 b. Were the pressure ulcers documented?
□ Yes □ No □ Don't know

14 a. Was the person's oral health assessed and documented at any time during the last week of life?
□ Yes □ No □ Don't know

If the answer is Yes, answer question 14B.
If the answer is No or Don't know, skip to question 15.

14 b. Was any disorder noted during assessment?
□ Yes □ No □ Don't know

15. Was anyone present at the time of death?
□ Yes, close friend(s) or relative(s)
□ Yes, close friend(s)/relative(s) and staff

□ Yes, staff
□ No
□ Don't know

16. Did the person's close friend(s)/relative(s) receive information about transition to end-of-life care, i.e. an individually tailored and informed conversation with a physician that is documented in the medical records about being in the final stage of life and about care being focused on quality of life and symptom relief?
□ Yes
□ Yes, offered but declined
□ No
□ Don't know
□ Had no close friend(s)/relative(s)

If the answer is Yes, No or Don't know, go to question 17.
If the answer is Had no close friend(s)/relative(s), skip to question 18.

17. Was/were the person's close friend(s)/relative(s) offered a follow-up talk within 1-2 months of the death?
□ Yes □ No □ Don't know

18. Did the person receive parenteral fluids/nutrition or enteral-tube feeding during the last 24 hours of life?
□ Yes □ No □ Don't know

19. Did the person display breakthrough of any of the following symptoms (19A-F) at any time during the last week of life?

19 a. Pain □ Yes □ No □ Don't know

If the answer is Yes, answer the following question.
If the answer is No or Don't know, skip to question 19B.

Pain was relieved: □ Completely □ Partially □ Not at all

19 b. Death rattle □ Yes □ No □ Don't know

If the answer is Yes, answer the following question.
If the answer is No or Don't know, skip to question 19C.

Death rattle was relieved: □ Completely □ Partially □ Not at all

19 c. Nausea ◻ Yes ◻ No ◻ Don't know

If the answer is Yes, answer the following question.
If the answer is No or Don't know, skip to question 19D.

Nausea was relieved: ◻ Completely ◻ Partially ◻ Not at all

19 d. Anxiety ◻ Yes ◻ No ◻ Don't know

If the answer is Yes, answer the following question.
If the answer is No or Don't know, skip to question 19E.

Anxiety was relieved: ◻ Completely ◻ Partially ◻ Not at all

19 e. Dyspnoea ◻ Yes ◻ No ◻ Don't know

If the answer is Yes, answer the following question.
If the answer is No or Don't know, skip to question 19F.

Dyspnoea was relieved: ◻ Completely ◻ Partially ◻ Not at all

19 f. Confusion ◻ Yes ◻ No ◻ Don't know

If the answer is Yes, answer the following question.
If the answer is No or Don't know, skip to question 20.

Confusion was relieved: ◻ Completely ◻ Partially ◻ Not at all

20. Was the person's pain assessed at any documented time during the last week of life using VAS, NRS or another pain-assessment tool?
◻ Yes ◻ No ◻ Don't know

21. Did the person experience severe pain at any time during the last week of life (e.g. VAS/NRS > 6 or severe pain according to another pain-assessment tool)?
◻ Yes ◻ No ◻ Don't know

22. Were the person's other symptoms assessed at any time during the last week of life using VAS, NRS or another symptom-assessment tool?
◻ Yes ◻ No ◻ Don't know

23. Was there an individual prescription of injectable PRN drugs on the drug list before death?

Opioids against pain	□ Yes	□ No	□ Don't know
Drugs against death rattle	□ Yes	□ No	□ Don't know
Drugs against nausea	□ Yes	□ No	□ Don't know
Drugs against anxiety	□ Yes	□ No	□ Don't know

24. How long before death was the person last examined by a physician?
□ Day/days
□ Week/weeks
□ Month or more
□ Don't know

25. Were specialists outside the team/ward consulted concerning the person's symptom relief during the last week of life (more than one answer option is possible)?
□ Yes, pain clinic
□ Yes, palliative-care team
□ Yes, other hospital unit
□ Yes, social worker/physiotherapist/occupational therapist/dietician
□ Yes, spiritual counsellor
□ No
□ Don't know

26. How satisfied is the team with the care delivered to the person during the last week of life?
□ 1 = Not at all □ 2 □ 3 □ 4 □ 5 = Completely

27. Date (year/month/day) of answering the questions ____________________

28. The questionnaire was answered by :
□ A single employee □ Staff jointly

29. Name and occupation of registrant_____________________________________
□ Physician □ Nurse □ Other staff

e-mail address ____
