# Supplementary material for: End-of-life care in hematological malignancies – a nationwide comparative study on the Swedish Register of Palliative Care
Source: PLoS One. 2025 Apr 29;20(4):e0312910. doi: 10.1371/journal.pone.0312910 (PMC12040083; doi:10.1371/journal.pone.0312910)
Supplement: S1 Table — All included variables from the end-of-life questionnaire, with frequencies of missing data and recoding information, when applicable. (DOCX) [file pone.0312910.s002.docx]

| **Variable** | **Available responses** | **Variable** | **Missing values**  **(Including ”Don’t know”)** | **Regrouping** |
| --- | --- | --- | --- | --- |
| Patient ID |  |  | No |  |
| Sex | Male/female | Demographic and confouding variable | No |  |
| Age |  | Demographic and confouding variable | No |  |
| Unit name |  |  | No |  |
| Date of death |  |  | No |  |
| The place of death is best described as: | 1. Own home (with support from specialized palliative care) 2. Own home (with support from general home care) 3. Own home (with support from in-home services) 4. Own home (without known support) 5. Nursing home – permanent stay 6. Nursing home – short term stay 7. Hospital: ward/patient facility/ICU (not hospice/palliative in-patient care) 8. Hospice/palliative in-patient care 9. Other | Outcome and grouping parameter | No | Yes,  Own home (a-d)  Nursing home (e+f)  Hospital (g)  In-patient palliative care (h)  Other (i)  For some analyses:  Specialised palliative care (a+h) |
| Disease/basic state that caused the death (more than one answer is possible): | a. Cancer  b. Cardiovascular disease  c. Respiratory disease  d. Cognitive disorder (dementia)  e. Stroke  f. Other neurological disease  g. Diabetes  h. Fracture  i. Multimorbidity  j. Infection  k. Other | Exclusion criteria | No |  |
| Based on the disease trajectory, was the death expected? | 1. Yes 2. No 3. Don’t know | Exclusion criteria | Total:  1,3%  Hematological:  2,6%  Solid tumors:  1,2% |  |
| Do the medical records include a documented decision by the physician responsible  to shift treatment/care to end-of-life care?* | 1. No 2. Yes, in free text 3. Yes, in classification code 4. Don’t know | Outcome parameter, communications | Total:  3,8%  Hematological:  3,3%  Solid tumors:  3,8%  Introduced in 2015, missing from all previous entries (11,7%) | Yes (b+c)  Not documented (a+d) |
| Did the person receive information about the transition to end-of-life care, i.e. an individually  tailored and informed conversation with a physician that is documented in the medical records about  being in the final stage of life | 1. Yes 2. No 3. No, lacks the ability to participate 4. No, offered but declined 5. No, guardian opposes 6. Don’t know | Outcome parameter, communications | Total:  9,1%  Hematological:  10,5%  Solid tumors:  9,0% | Offered (a+d)  No (b+e)  Unable to participate (c)  Unknown (d)  Included in statistical analysis:  Offered No |
| Was the persons preferred place to die known? | 1. Yes 2. No 3. Don’t know | Outcome parameter, communications | Total:  9,1%  Hematological:  11,3%  Solid tumors:  8,9% |  |
| Was anyone present at the time of death? | 1. Yes, close friend(s) or relative(s) 2. Yes, close friend(s) or relative(s) and staff 3. Yes, staff 4. No 5. Don’t know | Outcome parameter, communications | Total:  0,7%  Hematological:  1,0%  Solid tumors:  0,7% | Yes (a-c) |
| Did the person’s next of kin(s) receive information about transition to end-of-life care, i.e. an  individually tailored and informed conversation with a physician that is documented in the medical  records about being in the final stage of life? | 1. Yes 2. No 3. No, offered but declined 4. Had no close friend(s)/relative(s) 5. Had no known close friend(s)/ relative(s) 6. Don’t know | Outcome parameter, communications | Total:  6,7%  Hematological:  6,5%  Solid tumors:  6,7% | Offered (a+c)  No (b)  No known relations (d+e)  Included in statistical analysis:  Offered No |
| Was/were the person’s next of kin(s) offered a follow-up talk? | 1. Yes 2. No 3. Had no known close friend(s)/ relative(s) 4. Don’t know | Outcome parameter, communications | Total:  12,9%  Hematological:  17,5%  Solid tumors:  12,6% |  |
| Did the person receive parenteral fluids/nutrition during the last 24 hours of life? | 1. Yes 2. No 3. Don’t know | Outcome parameter, medical | Total:  0,5%  Hematological:  1%  Solid tumors:  0,5% |  |
| Did the person display breakthrough of any of the following symptoms at any time during  the last week of life?   1. Pain 2. Death rattle 3. Nausea 4. Anxiety 5. Dyspnoea 6. Confusion | 1. Yes 2. No 3. Don’t know | Outcome parameter,  symptom | Total:   1. 1,4% 2. 1,4% 3. 5,9% 4. 6,9% 5. 3,4% 6. 7,1%   Hematological:   1. 2,3% 2. 1,8% 3. 8,5% 4. 9,0% 5. 4,6% 6. 9,1%   Solid tumors:   1. 1,3% 2. 1,3% 3. 5,7% 4. 6,8% 5. 3,3% 6. 7,0% |  |
| If the answer was yes to the previous question, was the symptom relieved? | 1. Completely relieved 2. Partially relieved 3. Not at all relieved 4. Don’t know | Outcome parameter,  symptom | No | Not completely relieved (b+c) |
| Was the person’s pain assessed at any documented time during the last week of life using VAS,  NRS or another pain-assessment tool? | 1. Yes 2. No 3. Don’t know | Outcome parameter, medical | Total:  6,0%  Hematological:  7,4%  Solid tumors:  5,8% |  |
| Did the person experience severe pain at any time during the last week of life? | 1. Yes 2. No 3. Don’t know | Outcome parameter,  symptom | Total:  11,6%  Hematological:  14,4%  Solid tumors:  11,4% |  |
| Were the person’s other symptoms assessed at any time during the last week of life using VAS,  NRS or another symptom-assessment tool? | 1. Yes 2. No 3. Don’t know | Outcome parameter, medical | Total:  9,1%  Hematological:  10,9%  Solid tumors:  9,0% |  |
| Was there an individual prescription of injectable PRN drugs on the drug list before death?   1. Opioids against pain 2. Drugs against death rattle 3. Drugs against nausea 4. Drugs against anxiety | 1. Yes 2. No 3. Don’t know | Outcome parameter, medical | Total:   1. 0,2% 2. 0,4% 3. 0,8% 4. 0,4%   Hematological:   1. 0,3% 2. 0,8% 3. 1,3% 4. 0,8%   Solid tumors:   1. 0,2% 2. 0,4% 3. 0,7% 4. 0,4% |  |
| Were specialists outside the team/ward consulted concerning the person’s symptom relief during  the end of life (more than one answer option is possible)? | 1. Yes, pain clinic 2. Yes, palliative-care team 3. Yes, other hospital unit 4. Yes, social worker/ physiotherapist/ occupational therapist/ dietician 5. Yes, spiritual counsellor 6. No 7. Don’t know | Outcome parameter, medical | Total:  2,6%  Hematological:  4,1%  Solid tumors:  2,5% |  |
