# Supplementary material for: End-of-life care in hematological malignancies – a nationwide comparative study on the Swedish Register of Palliative Care
Source: PLoS One. 2025 Apr 29;20(4):e0312910. doi: 10.1371/journal.pone.0312910 (PMC12040083; doi:10.1371/journal.pone.0312910)
Supplement: S2 Table — Place of death and access to specialized palliative care for all hematological diagnoses, with lymphoma subgroups. (DOCX) [file pone.0312910.s003.docx]

**S2 Table. Place of death and SPC access, all hematological diagnoses**

|  | **ALL (N=190)** | **AML (N=1804)** | **CLL (N=539)** | **CML (N=131)** | **Aggressive B-cell lymphoma (N=648)** | **Hodgkin lymphoma (N=104)** | **Indolent B-cell lymphoma (N=2078)** | **T/NK-cell lymphoma (N=262)** | **MDS (N=549)** | **MPN (N=138)** | **Myeloma (N=1956)** | **NOS* (N=151)** |
| --- | --- | --- | --- | --- | --- | --- | --- | --- | --- | --- | --- | --- |
| **Age** | | | | | | | | | | | | |
| Median  [range] | 71 [18,96] | 76  [19,98] | 79  [21,99] | 76 [23,97] | 75  [21,97] | 76  [18,93] | 78 [24,101] | 73.5 [18,97] | 79  [37,97] | 78 [29,99] | 75  [21,99] | 80 [18,103] |
| **Place of death** | | | | | | | | | | | | |
| Own home | 46  (24 %) | 422  (23 %) | 101  (19 %) | 33  (25 %) | 138  (21 %) | 12  (12 %) | 394  (19 %) | 56  (21 %) | 118  (21 %) | 26  (19 %) | 400  (20 %) | 35  (23 %) |
| Nursing home | 12  (6 %) | 128  (7 %) | 90  (17 %) | 16  (12 %) | 76  (12 %) | 21  (20 %) | 370  (18 %) | 24  (9 %) | 72  (13 %) | 27  (20 %) | 284  (15 %) | 41  (27 %) |
| Emergency hospital | 75  (39 %) | 771  (43 %) | 233  (43 %) | 41  (31 %) | 243  (38 %) | 39  (38 %) | 709  (34 %) | 101  (39 %) | 201  (37 %) | 49  (36 %) | 743  (38 %) | 37  (25 %) |
| Hospice / palliative  in-patient care | 55  (29 %) | 472  (26 %) | 114  (21 %) | 40  (31 %) | 187  (29 %) | 32  (31 %) | 593  (29 %) | 81  (31 %) | 156  (28 %) | 34  (25 %) | 520  (27 %) | 37  (25 %) |
| Other | 2  (1 %) | 11  (1 %) | 1  (0 %) | 1  (1 %) | 4  (1 %) | 0  (0 %) | 12  (1 %) | 0  (0 %) | 2  (0 %) | 2  (1 %) | 9  (0 %) | 1  (1 %) |
| **Access to specialized palliative care** | | | | | | | | | | | | |
| In specialized palliative unit^#^ | 91  (48 %) | 804  (45 %) | 175  (32 %) | 62  (47 %) | 289  (45 %) | 39  (38 %) | 862  (41 %) | 120  (46 %) | 247  (45 %) | 55  (40 %) | 791  (40 %) | 58  (38 %) |
| Other^§^, with  palliative team consulted | 23  (12 %) | 161  (9 %) | 38  (7 %) | 11  (8 %) | 78  (12 %) | 9  (9 %) | 198  (10 %) | 33  (13 %) | 37  (7 %) | 10  (7 %) | 202  (10 %) | 12  (8 %) |
| None | 76  (40 %) | 839  (47 %) | 326  (60 %) | 58  (44 %) | 281  (43 %) | 56  (54 %) | 1018  (49 %) | 109  (42 %) | 265  (48 %) | 73  (53 %) | 963  (49 %) | 81  (54 %) |
| * Hematological malignancy, not otherwise specified ^#^ Hospice / palliative in-patient care OR Own home with support of specialized palliative team ^§^Any place of death apart from specialized palliative unit | | | | | | | | | | | | |
