# Supplementary material for: End-of-life care in hematological malignancies – a nationwide comparative study on the Swedish Register of Palliative Care
Source: PLoS One. 2025 Apr 29;20(4):e0312910. doi: 10.1371/journal.pone.0312910 (PMC12040083; doi:10.1371/journal.pone.0312910)
Supplement: S3 Table — Frequencies of co-morbidities in initial SRPC dataset of expected deaths. (DOCX) [file pone.0312910.s004.docx]

**S Table 3. Frequencies of co-morbidities**

|  | **Hematological malignancy (N=13427)** | **Solid tumor (N=139458)** | **P-value** |
| --- | --- | --- | --- |
| **Cardiac disease** | | | |
| Yes | 1590 (12 %) | 9374 (7 %) | <0.001 |
| No | 11837 (88 %) | 130084 (93 %) |  |
| **Respiratory disease** | | | |
| Yes | 555 (4 %) | 6773 (5 %) | <0.001 |
| No | 12872 (96 %) | 132685 (95 %) |  |
| **Cognitive disorder (dementia)** | | | |
| Yes | 598 (4 %) | 4262 (3 %) | <0.001 |
| No | 12829 (96 %) | 135196 (97 %) |  |
| **Stroke** | | | |
| Yes | 356 (3 %) | 2300 (2 %) | <0.001 |
| No | 13071 (97 %) | 137158 (98 %) |  |
| **Other neurological disease** | | | |
| Yes | 0 (0 %) | 0 (0 %) | NS |
| No | 13308 (99 %) | 138639 (99 %) |  |
| Missing | 119 (0.9%) | 819 (0.6%) |  |
| **Diabetes** | | | |
| Yes | 292 (2 %) | 2637 (2 %) | 0.0239 |
| No | 13135 (98 %) | 136821 (98 %) |  |
| **Fracture** | | | |
| Yes | 101 (1 %) | 819 (1 %) | 0.0213 |
| No | 13326 (99 %) | 138639 (99 %) |  |
| **Infection** | | | |
| Yes | 320 (2 %) | 991 (1 %) | <0.001 |
| No | 13107 (98 %) | 138467 (99 %) |  |
| **Multimorbidity** | | | |
| Yes | 1997 (15 %) | 9120 (7 %) | <0.001 |
| No | 11430 (85 %) | 130338 (93 %) |  |
| **Other comorbidities** | | | |
| Yes | 1059 (8 %) | 3493 (3 %) | <0.001 |
| No | 12368 (92 %) | 135965 (97 %) |  |

Frequencies of co-morbidities in the original SRPC dataset of expected deaths.
