# Supplementary material for: End-of-life care in hematological malignancies – a nationwide comparative study on the Swedish Register of Palliative Care
Source: PLoS One. 2025 Apr 29;20(4):e0312910. doi: 10.1371/journal.pone.0312910 (PMC12040083; doi:10.1371/journal.pone.0312910)
Supplement: Attachment — Submitted filename: Response_to_reviewers.docx [file pone.0312910.s006.docx]

Response to reviewers and editor

Thank you for considering this paper for publication, and for the encouraging comments. We have tried to address all the issues raised, as detailed below. Our responses are marked in red.

First some responses to the editor, please let us know if anything is still unclear.

Role of funder: Blodcancerfonden as a funding agent had no role in study design, data collection and analysis, decision to publish, or preparation of the manuscript. Patient representatives mediated by Blodcancerfonden were involved in study design.

Regarding data sharing, we are unable to share the data due to the following reason as stated on submission:

The data contain potentially identifying information regarding individuals and therefore are subject to ethical and legal restriction to public sharing. We cannot share the data set as it is not permitted by Swedish law and the ethical permission obtained only allows for public sharing on a group level. Data are available from the Swedish Register of Palliative Care (info@palliativregistret.se) for researchers after appropriate ethical review.

Responses to reviewer 2:

Firstly in general regarding the conclusions and results; I think it would be better to "soften" the headings and the language describing the results/conclusions. Can we really say that an outcome is "impacted" by something? I would say "associated with" and/or correlated with depending on what analysis is done- we can not be sure that for example having a hematological diagnosis impacts anything, but is associated with. Throughout the whole manuscript you speak of impact- that is to me to draw the conclusions a bit far. I do understand what you mean, but a more strict language would be appropriate, since you show associations between for example death in an emergency hosptial and worse symptoms.

*We agree, and have changed the wording in the manuscript to tone down the implications.*

Another suggestion is to be more clear with you analysis and what has been done?
1. the Swedish palliative register - this is the last week in life? or how long back?

*Yes, if not otherwise specified the variables are recorded for the last 7 days of life. This is now specified in the Methods section, row 112-113.*

2. Please clarify in methods sections what co-morbidities was classified as exclusion criteria?

*This is now included in Methods, row 103-105*

3. What were the dependent variables? Did you do a multivariate (several dependent variables) or a multiple regression analyis (one dependent variable)? It is very hard to follow how you did the analysis, which also leads to difficulties in interpreting the results.

*The method used was multiple regression analysis, using the glm() function in R with the outcome variable as dependent variable. This is now clarified in the Methods section, row 119.*

In line 104 there seems to be a word missing or a comma/period in the wrong place- not sure what is meant?

*This is a typing error and has been removed.*

In line 214 in results section- here it is very unclear how this was done? did you add this variable as a co-variate in the analysis-perhaps just how it is written.

*In this analysis we included iv fluids as a variable in the multiple regression analysis, in addition to age, sex and HM/ST. This has been clarified in the section.*

I would like a reasoning on excluding unexpected deaths and co-morbidities? I think this is the real situation caring for patients with hematological malignancies, that sometimes death is quick and unexpected, both due to the disease itself, and also the treatment. Maybe clarify why not include also these patients?

In line 266 in discussion, you draw the conclusion/reflection that since there was a documented end-of-life conversation, it could be a sign that they did not dy of curative treatment. But the line between curative and not is very fine in hematological patients, and it might be considered a curative treatment even if very slim chance- so not sure I follow this line of reasoning.
Another comment is that it could be that the unexpected deaths (that is excluded in you study), would be patients whith a very slim chance of cure, that died during or in complications of treatment, were totally "ignored" in terms of palliative needs, and this may be to a large extent impacting your results- so wish a more in depth method discussion on the choice to exclude these patients?(if I understood the meaning of an unexpected death in the right way)?

*Yes, this is very likely and we agree that the palliative needs of the hematological patients is probably under-estimated in this study. We have aimed for a conservative estimate, focusing on the patients where the conditions could be expected to be “optimal” for identifying and attending to palliative needs (expected deaths and no co-morbidities) as the nature of hematological diseases with “quick and unexpected” deaths are often used as an explanation for why hematological patients are less likely to be referred to palliative care. As we could show that even under optimal circumstances (as far as such can be identified in registry data) there are significant differences between hematological and solid tumor patients, we hope that this study can lead to discussions and possibly changes in practice in hematological care.*

*For unexpected deaths, the SPRC did not collect most of the variables of interest during the study period, and these cases were therefore not possible to include for most of the analyses.*

Furhter, line 295-7 in the discussion, is this really the only explanation? could it not be that for example pain assessment or oral assessments are done but not documented (thus not captured when registering in register?)- can we really assume that the missing data cohort have poorer quality of care at end of life? I agree with your reflections, but would be good to mention these institutions that do not even register in this registry?

*Yes, it is definitely possible, even likely, that assessments have been done but not documented. But that would happen in both HM and ST patients. This section in the discussion is only addressing the question whether the missing data is likely to be causing the significant differences reported, which would be the case if missing data could be expected to correlate with a higher likelihood of having made symptom assessments. We find this to be unlikely.*

I am overall in the discussion missing the reasoning on how to integrate palliative care in this group. We need to hava a palliative approach already during the treatment, and pursue both lines simultaneously- that is even a more challenge in my experience, and if we are going to provide high quality of end-of-life care for hematological patients in an emergency hospital, we need to talk about this. If a small chance of cure, what do we do and plan and hope for if this does not work? The solution can not be that as long as patients get palliative home care or hospice all is good.

*This is a very important point, and a discussion larger than this particular study. We have expanded the discussion in this regard, and hope for a continuation of that discussion in other forums.*

In summary I welcome this important work, and with some clarifications this will be a good contribution to the body of research on patients with hematological malignancies and the need of palliative care/approach.
